# The comparison of dexmedetomidine and midazolam premedication on postoperative anxiety in children for hernia repair surgery: A randomized controlled trial

**DOI:** 10.1111/pan.13667

**Published:** 2019-07-03

**Authors:** Zhen Du, Xi‐Ying Zhang, Shuang‐Quan Qu, Zong‐Bing Song, Si‐Wei Wei, Zhen Xiang, Qu‐Lian Guo

**Affiliations:** ^1^ Department of Anesthesiology Xiangya Hospital of Central South University Changsha China; ^2^ Department of Anesthesiology Hunan Children' Hospital Changsha China

**Keywords:** anxiety, pediatrics, perioperative period, surgery

## Abstract

**Background:**

Perioperative anxiety is common in pediatric patients undergoing surgery.

**Aims:**

The aim of this study was to determine whether an infusion of dexmedetomidine prior to hernia repair in children provides better postoperative anxiety outcomes that a preoperative infusion of midazolam.

**Methods:**

Ninety 6‐11‐year‐old children, who were scheduled to undergo elective hernia repair, were enrolled for this double‐blind, randomized controlled trial. Group D (n = 45) received an intravenous infusion of dexmedetomidine (0.5 μg/kg) and Group M (n = 45) received an intravenous infusion of midazolam (0.08 mg/kg) in 20 mL of normal saline for 10 minutes before the induction of anesthesia. Pre‐ and postoperative scores on the modified Yale Preoperative Anxiety Scale were the main outcomes. Secondary outcomes included systolic blood pressure, diastolic blood pressure, heart rate, and postoperative pain measured on a visual analogue scale and patient satisfaction using a numerical rating scale.

**Results:**

Postoperative anxiety in Group D was significantly lower than preoperative anxiety (2 hours postoperatively mean difference [95% CI]: 2.83 [0.87‐4.79], *P* = 0.036, 4 hours postoperatively mean difference [95% CI]: 3.29 [1.39‐5.20], *P* = 0.005). Preoperative and postoperative anxiety in Group M was similar. Anxiety scores in Group D were also significantly lower than anxiety in Group M 2 hours (mean difference [95% CI]: 1.89 [0.52‐3.26], *P* = 0.01) and 4 hours (mean difference [95% CI]: 3.32 [1.98‐4.66], *P* < 0.001) postoperatively. Systolic blood pressure, diastolic blood pressure and heart rate were lower in Group D than in Group M after administration of sedative drugs until children left PACU (SBP mean difference [95% CI]: 13.87 [10.30‐17.43], *P* < 0.001, DBP mean difference [95% CI]: 5.96[3.80‐8.11], *P* < 0.001, HR mean difference [95% CI]: 10.36 [7.58‐13.13], *P* < 0.001). Pain was also significantly lower in Group D than in Group M at 2 hours (median difference [95% CI]: 1 [0.26‐1.34], *P* = 0.004), 4 hours (median difference [95% CI]: 1 [0.31‐1.02], *P* = 0.003), and 1 day (median difference [95% CI]: 0 [0.22‐0.76], *P* = 0.003) postoperatively. Patient satisfaction scores were significantly higher in Group D than in Group M 1 day (median difference [95% CI]: 0 [−0.83 to −0.24], *P* = 0.006) and somewhat higher 1 week (median difference [95% CI]: 0 [−0.67 to −0.04], *P* = 0.06) postoperatively.

**Conclusion:**

Compared with midazolam, a single preoperative intravenous dose of dexmedetomidine appears to provide better postoperative anxiolytic effects for children undergoing same‐day surgery.

**What is already known**

- Dexmedetomidine premedication effectively decreases children's preoperative anxiety.

**What this article adds**

- Dexmedetomidine premedication effectively decreases children's postoperative anxiety.

## 1 INTRODUCTION

The perioperative period is a stressful time for most patients undergoing surgery, especially children. It is estimated that 50%‐75% of children undergoing surgery experience significant anguish and anxiety throughout the entire perioperative period.^1^ Anxiety can lead to negative behavior manifestations postoperatively, with 54% of children exhibiting negative behavior manifestations 2 weeks postsurgery and 20% continuing to show them for 6 months.^2^

Numerous studies have examined the effects of various factors on postoperative anxiety and negative behavior manifestations. These factors include the child's and parents' preoperative anxiety, the child's temperament, previous hospitalization for surgery, length of hospitalization, preoperative preparation and medications, the kind of anesthetic drugs used, the presence of parents during the induction of anesthesia, and the child's experience in the postanesthetic care unit (PACU).^2^, ^3^, ^4^, ^5^, ^6^, ^7^

It is imperative to investigate ways to address perioperative anxiety, especially as some pediatric patients display significant postoperative anxiety.^8^ The administration of anxiolytic premedication has focused on patients' preoperative stress, but postoperative recovery has become increasingly important and should be considered when anesthesiologists decide to prescribe premedication.^9^ A study by LaMontagne et al concluded that improved medication management during the preoperative period should achieve better outcomes in young patients including decreased preoperative and postoperative anxiety.^10^

The most common medication for reducing preoperative anxiety in children is midazolam, which has a rapid onset and limited duration of action.^11^ However, midazolam is not an ideal drug because it has several undesirable effects, including postoperative behavioral changes, restlessness, cognitive impairment, paradoxical reactions, and respiratory depression.^12^ Moreover, it is ineffective in preventing emergence agitation (EA) and emergence delirium (ED) in children.^13^ Therefore, more appropriate preoperative medications are needed to improve the perioperative safety and comfort of children.

Dexmedetomidine is a highly specific a_2_‐adrenergic receptor agonist that has numerous applications. Studies have demonstrated that dexmedetomidine premedication effectively decreases children's separation anxiety, promotes the acceptance of mask induction,^14^, ^15^ and provides good postoperative analgesia, which reduces the need for opioids.^16^, ^17^ A single intravenous (IV) injection of dexmedetomidine before surgery also decreases the frequency of EA and ED during the first 2 hours postoperatively.^18^, ^19^ However, the postoperative anxiolytic effects of dexmedetomidine have not been examined in pediatric patients.

We hypothesized that in children undergoing outpatient surgery, a single IV dose of dexmedetomidine before anesthesia would be associated with less postoperative anxiety than a single dose of midazolam.

## 2 MATERIALS AND METHODS

### 2.1 Patient selection

We conducted a double‐blind, randomized controlled trial (RCT) at Hunan Children's Hospital in China. Written informed consent was obtained from parents and verbal consent was obtained from each child.

A total of 90 6‐11‐year‐old children were enrolled in the study, who had an ASA physical status I or II and were scheduled for elective hernia repair between September 2017 and December 2017 (trial registry identifier, ChiCTR‐IPR‐17012920). The exclusion criteria were: complicated hernia, a known allergy or hypersensitive reaction to dexmedetomidine or midazolam, long‐term use of sedative or analgesic drugs, a past history of surgery or anesthesia, current upper respiratory infections, asthma, developmental delay, psychological diagnosis requiring active treatment, or congenital or neurological diseases (Appendix S2).

### 2.2 Study design

The children were randomly assigned to two groups using a computer‐generated random numbers table: Group M (n = 45) and Group D (n = 45). An anesthesia nurse not involved in the experiment prepared the drugs, and the surgeons, anesthetists, children, and parents were blind to group allocation. Operations were performed by a surgical team that included three pediatric surgeons (Appendix S1).

Pain was measured after surgery with a visual analogue scale (VAS), ranging from 0 (no discomfort and no pain) to 10 (high discomfort and maximum pain). Patient satisfaction was measured after surgery using a numerical rating scale (NRS), ranging from 0 (least satisfied) to 10 (most satisfied). Both scales were explained to the children and their parents during a preoperative visit.

All children followed the ASA fasting guidelines.^20^ On the day of surgery, an IV line was inserted under local anesthesia while the child was in a hospital ward; the child went to a preanesthesia holding room approximately 20 minutes before surgery, accompanied by one parent. Prior to receiving premedication, blood pressure, ECG, oxygen saturation (SPO_2_), and heart rate (HR) were measured and monitored continuously.

Children in Group M received an IV infusion of midazolam (0.08 mg/kg) and children in Group D received an IV infusion of dexmedetomidine (0.5 μg/kg), both in 20 mL of normal saline, for 10 minutes. The children were carried to the operating room (OR) under deep sedation.

### 2.3 Anesthesia and analgesia procedures

All anesthesia procedures were performed by the same two attending anesthesiologists using sufentanyl 0.2 μg/kg, inhalation of sevoflurane (4%‐6%) in oxygen at 6 L/min for induction, and IV injections of 0.01 mg/kg phencyclidine.

A laryngeal mask airway was placed after induction with spontaneous ventilation maintained. Carbon dioxide partial pressure (P_ET_CO_2_) was monitored. If P_ET_CO_2_ reached 60 mm Hg and SpO_2_ decreased, assisted ventilation was given. Anesthesia was maintained by inhalation of oxygen‐sevoflurane 2 L/min, which was regulated according to the depth of anesthesia, and monitored throughout the operation to keep the Narctrend index between 40 and 60.

Lactated Ringer's solution (10 mL/kg/h) was administered intraoperatively for fluid maintenance. The intraoperative HR and MAP were controlled within a 30% variation around the baseline levels with atropine and vasoactive drugs.

The inhalation of sevoflurane was stopped and IV infusion of sufentanyl was administered at a dose of 0.05 μg/kg/h for postoperative analgesia after surgery. The laryngeal mask was removed in the OR when P_ET_CO2 ≤ 50 mm Hg.

The children were transferred to the PACU (which took <1 minute in all cases), where they were monitored. When the Aldrete score reached 10 (full recovery from sedation), they were discharged from the PACU and transferred to the ward. The children were discharged from the hospital within 24 hours.

### 2.4 Monitoring hemodynamic changes

Systolic blood pressure (SBP), diastolic blood pressure (DBP), and HR were recorded at seven time points: T0 = prior to midazolam/dexmedetomidine infusion; T1 = 5 minutes after transfer to the OR; T2 = 5 minutes after laryngeal mask insertion; T3 = 5 minutes after surgery initiation; T4 = 5 minutes after laryngeal mask removal; T5 = 5 minutes after transfer to the PACU; and T6 = at PACU discharge.

### 2.5 Assessment of anxiety

The modified Yale Preoperative Anxiety Scale (m‐YPAS)^21^ was used to assess preoperative and postoperative anxiety. The m‐YPAS is a validated observational tool for assessing children's anxiety, which includes five items: activity, emotional expressivity, state of arousal, vocalization, and use of parents. Each item has four categories, except vocalization, which has six categories. A partial score is allocated to each item, and the sum is divided by the number of categories in that item. The item scores are summed and the sum is multiplied by 20. Scores of 23.5‐30 indicate no or mild anxiety, whereas scores >30 indicate severe anxiety.^22^

An anesthesiologist, blind to group assignment, assessed anxiety during a preoperative interview, 5 minutes after drug administration (in the preanesthesia holding room), before the child was brought to the OR, and 2 hours and 4 hours postoperatively. All research team members were trained in data collection and scoring the tools to ensure inter‐rater reliability.

### 2.6 Obtaining postoperative VAS and NRS scores

The anesthesiologist who assessed anxiety also recorded children's VAS scores 2 hours and 4 hours postoperatively. The VAS and NRS scores at 1 day, 1 week, and 1 month postoperatively were obtained by telephone follow‐up.

### 2.7 Statistical analyses

The data are expressed as the mean ± standard deviation (SD), median, interquartile range (IQR), or numbers. The m‐YPAS, VAS, and NRS scores were analyzed using the Kruskal‐Wallis test. Age, weight, surgery duration, HR, SBP, and DBP were analyzed using two‐sample *t*‐tests. Gender was analyzed by the χ^2^ test. Statistical analyses were performed using SPSS 19.0 (SPSS, Institute). *P‐*values < 0.05 were considered statistically significant.

## 3 RESULTS

### 3.1 Children's characteristics

Table 1 shows the characteristics of the study participants. There were no significant differences in the demographics and the duration of the operations between the two groups. All patients were discharged as scheduled without any complications.

**Table 1:** Characteristics of the children in Groups M and D (demographics and duration of surgery)

Variables	Group M (n = 45)	Group D (n = 45)
Age (y)	7.6 ± 1.60	7.5 ± 1.55
Males/females	24/21	26/19
Body weight (kg)	25.48 ± 5.42	24.89 ± 6.60
Duration of surgery(min)	24.29 ± 5.02	25.80 ± 6.69

Note

Data shown are the number or mean ± SD.

Abbreviations: D, dexmedetomidine; M, midazolam.

### 3.2 Hemodynamic variations

**Figure 1:**
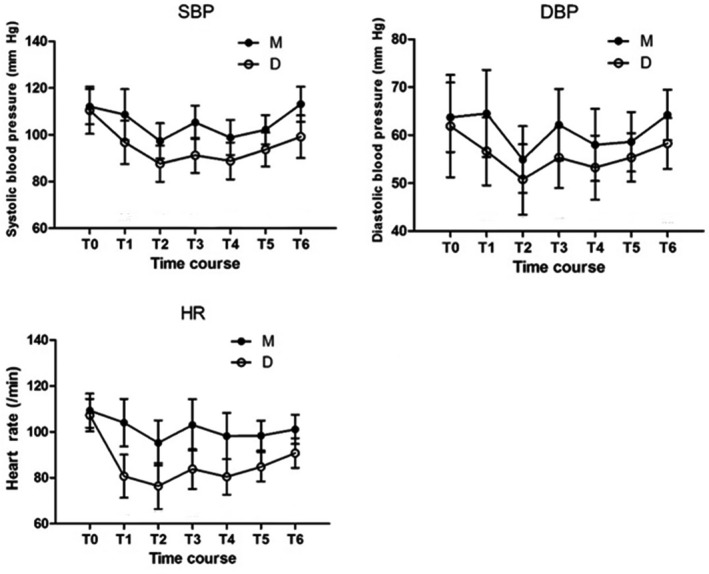
Hemodynamic variations

Figure 1 shows the preoperative hemodynamics of the two groups were similar at baseline, and decreased significantly in both groups after administration of sedative drugs. The decrease was significantly greater in Group D than Group M (SBP mean difference [95% CI]: 11.87 [7.73‐16.00],* P* < 0.001, DBP mean difference [95% CI]:7.87 [4.43‐11.30], *P* < 0.001, HR mean difference [95% CI]: 23.29 [18.64‐27.93], *P* < 0.001). The blood pressure and HR in group D were lower than those in group M during surgery and after surgery until children left PACU (SBP mean difference [95% CI]: 13.87 [10.30‐17.43],* P* < 0.001, DBP mean difference [95% CI]: 5.96[3.80‐8.11], *P* < 0.001, HR mean difference [95% CI]: 10.36 [7.58‐13.13], *P* < 0.001). No incidence of cardiovascular instability requiring an intervention occurred in any of the patients.

### 3.3 Anxiety

The children's m‐YPAS scores are shown in Table 2.

**Table 2:** Modified Yale Preoperative Anxiety Scale (M‐YPAS) scores, Vas pain scores, patient satisfaction scores, and postoperative inflammation factors

Variables	Group M (n = 45)	Group D (n = 45)	Mean/median difference (95% CI）	*P* value
M‐YPAS scores				
Preoperative interview	27.88 ± 3.34	28.15 ± 6.38	−0.27 (−2.29 to 1.75)	0.28
5 min after drug administration	29.29 ± 4.34	32.71 ± 9.99	−3.42 (−6.56 to −0.27)	0.09
After transfer to the OR	29.36 ± 6.86	27.09 ± 5.20	2.26 (−0.03 to 4.56)	0.06
2 h postoperative	27.21 ± 3.70	25.32 ± 2.65	1.89 (0.52 to 3.26)	0.01
4 h postoperative	28.18 ± 4.02	24.86 ± 2.35	3.32 (1.98 to 4.66)	<0.001
Vas pain scores				
2 h postoperative	1 (2)	0 (1)	1 (0.26 to 1.34)	0.004
4 h postoperative	1 (2)	0 (1)	1 (0.31 to 1.02)	0.003
1 d postoperative	0 (1)	0 (0)	0 (0.22 to 0.76)	0.003
1 wk postoperative	0 (0)	0 (0)	0 (0.01 to 0.39)	0.28
1 mo postoperative	0 (0)	0 (0)	0 (−0.04 to 0.35)	0.13
Patient satisfaction scores				
1 d postoperative	9 (2)	10 (1)	0 (−0.83 to −0.24)	0.006
1 wk postoperative	10 (1)	10 (1)	0 (−0.67 to −0.04)	0.06
1 mo postoperative	10 (1)	10 (1)	0 (−0.54 to 0.09)	0.29

Note

Data shown are the number or mean ± SD.

Abbreviations: D, dexmedetomidine; M, midazolam.

**Figure 2:**
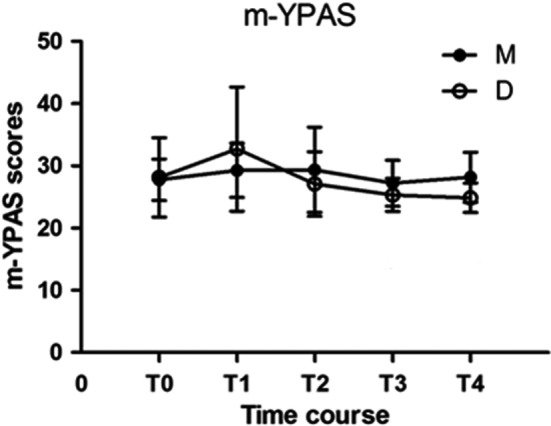
Perioperative anxiety scores

Figure 2 shows the two groups had comparable, mild anxiety, preoperatively (T0). There was no statistically significant difference in preoperative and postoperative anxiety (2 hours mean difference [95% CI]: 0.67 [−0.47 to 1.82], *P* = 0.25), 4 hours mean difference [95% CI]: −0.30 [−1.48 to 0.88], *P* = 0.81) in group M. Anxiety increased in Group D 5 minutes after drug administration (T1), but subsequently decreased. Anxiety at 2 hours (mean difference [95% CI]: 2.83 [0.87‐4.79], *P* = 0.036), 4 hours (mean difference [95% CI]: 3.29 [1.39‐5.20], *P* = 0.005) postoperatively was significantly lower than anxiety preoperatively in Group D. Anxiety was also significantly lower in Group D than in Group M 2 hours (mean difference [95% CI]: 1.89 [0.52‐3.26], *P* = 0.013) and 4 hours (mean difference [95% CI]: 3.32 [1.98‐4.66], *P* < 0.001) postoperatively.

### 3.4 Pain and satisfaction

The children's pain scores and satisfaction scores are shown in Table 2.

**Figure 3:**
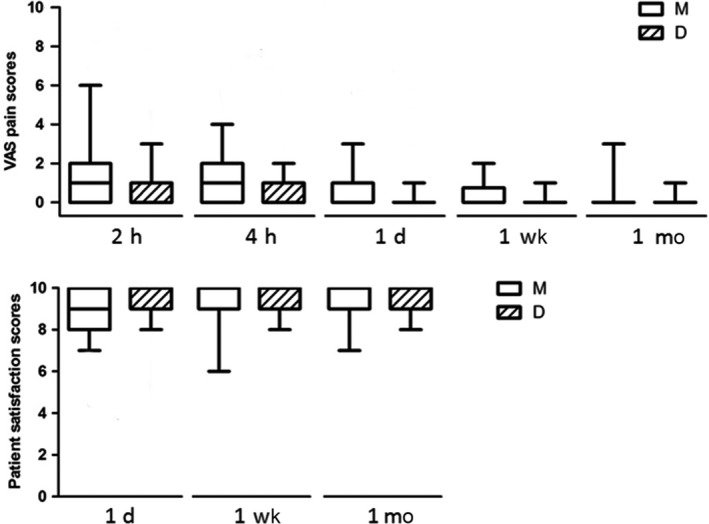
Postoperative Vas scores and patient satisfaction scores

Figure 3 shows pain scores were much lower for Group D than Group M at 2 hours (median difference [95% CI]: 1 [0.26‐1.34], *P* = 0.004), 4 hours (median difference [95% CI]: 1 [0.31‐1.02], *P* = 0.003), and 1 day (median difference [95% CI]: 0 [0.22‐0.76], *P* = 0.003) postoperatively. Then, pain in both groups decreased and was minimal by postsurgical week‐1, with no group difference. Children in Group D were significantly more satisfied than those in Group M (median difference [95% CI]: 0 [−0.83 to −0.24], *P* = 0.006) 1 day postoperatively and somewhat more satisfied (median difference [95% CI]: 0 [−0.67 to −0.04], *P* = 0.06) 1 week postoperatively.

## 4 DISCUSSION

This study was undertaken to evaluate the use of a single dose of dexmedetomidine, preoperatively, to relieve postoperative anxiety in children undergoing surgery. Children given preoperative dexmedetomidine had significantly lower anxiety in the postoperative period, compared with those given midazolam.

A meta‐analysis of RCTs concluded that dexmedetomidine effectively decreased separation anxiety and postoperative agitation, and provided more effective postoperative analgesia than midazolam.^16^ This hypothesis of this study was based on the fact that dexmedetomidine has a half‐life of 2‐3 hours,^23^ so its analgesic effects are likely to persist into the recovery period,^24^ thereby reducing the postoperative anxiety of children undergoing surgeries that take a relatively short amount of time.

Anxiety increased in Group D 5 minutes after drug administration (T1), but decreased by the time children had been transferred to the OR. The onset time of dexmedetomidine is 10‐15 minutes and it takes nearly 30 minutes to reach its peak concentration may be the reason. Pre‐ and postoperative anxiety in Group M was similar, while postoperative anxiety in Group D was significantly lower than preoperative anxiety in Group D and postoperative anxiety in Group M. Hence, dexmedetomidine had a postoperative anti‐anxiety effect, while midazolam did not.

The dexmedetomidine dose in this study was selected in light of previous findings that infusion of dexmedetomidine ≧0.5 μg/kg before surgery significantly decreases the frequency of EA and ED postoperatively^18^, ^19^; the dose of midazolam followed the standard hospital protocol. From the result there was no difference between the two groups in alleviating separation anxiety, the dose of midazolam for this comparison was correct. We found that 0.5 μg/kg of dexmedetomidine reduced postoperative anxiety and produced no serious hemodynamic events. The relatively low dose may explain the absence of adverse hemodynamic events. The drug's effectiveness to reduce postoperative anxiety may improve at higher doses, but higher doses could increase its side effects.

After sedation, blood pressure and heart rate decreased significantly in both groups. When the children were fully awake and discharged from the PACU, the blood pressure and HR in group D were significantly lower than those in group M, which could be due to direct dexmedetomidine effect or less anxiety.

The pain scores of the dexmedetomidine group were lower than those of the midazolam group at 2 hours, 4 hours, and 1 day postoperatively, but there were no differences, 1 week and 1 month postoperatively. Children in dexmedetomidine group were more satisfied 1 day and somewhat more satisfied 1 week postoperatively than children in the midazolam group. Anxiety decreases when there is an increase in satisfaction.^25^ A comparison of satisfaction and pain scores on 1 week postoperatively indicates that the difference in scores may be related to anxiety besides pain and there may be other factors affecting postoperative anxiety besides pain.

## 5 STUDY LIMITATIONS

This study has several limitations. First, the child's temperament and parental demographic, psychosocial, and other variables that can affect postoperative anxiety, were not assessed. The second is the timing and dose of drug administration. Dexmedetomidine probably did not reach its peak effect when the children entered the OR and the midazolam dose might not have been equipotent to the dexmedetomidine dose. Third, we used the m‐YPAS, which is not validated for postoperative anxiety in children, as no such validated scale is available. Moreover, anxiety was only assessed twice postoperatively and these assessments might not have reflected the children's long‐term status.

## 6 CONCLUSION

Our study demonstrates that dexmedetomidine, compared with midazolam, as premedication, can lower postoperative anxiety in children undergoing same‐day surgery.

## CONFLICT OF INTEREST

The authors report no conflicts of interest.

## ETHICAL APPROVAL

The study protocol was approved by the Research and Ethics Committee of Hunan Children's Hospital on September 25, 2016. The approval code number was HCHLL‐2016‐002.
