# Supplementary material for: The comparison of dexmedetomidine and midazolam premedication on postoperative anxiety in children for hernia repair surgery: A randomized controlled trial
Source: Paediatr Anaesth. 2019 Jul 3;29(8):843–9. doi: 10.1111/pan.13667 (PMC6852055; doi:10.1111/pan.13667)
Supplement: Supplementary file 2 [file PAN-29-843-s002.doc]

**
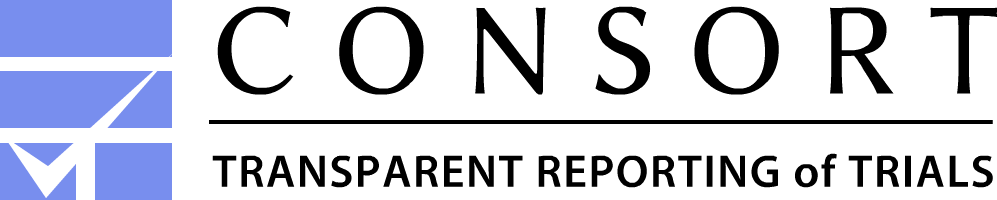
**

**CONSORT 2010 Flow Diagram**

**Allocation**

**Analysis**

**Follow-Up**

**Enrollment**

Assessed for eligibility (n=90 )

Excluded (n= 0 )

♦  Not meeting inclusion criteria (n=0 )

♦  Declined to participate (n=0 )

♦  Other reasons (n= 0 )

Analysed (n=45)
♦ Excluded from analysis (give reasons) (n= 0)

Lost to follow-up (give reasons) (n= 0)

Discontinued intervention (give reasons) (n= 0)

Allocated to intervention (n= 45)

♦ Received allocated intervention (n=45)

♦ Did not receive allocated intervention (give reasons) (n=0)

Lost to follow-up (give reasons) (n= 0)

Discontinued intervention (give reasons) (n= 0)

Allocated to intervention (n=45)

♦ Received allocated intervention (n=45)

♦ Did not receive allocated intervention (give reasons) (n=0)

Analysed (n= 45)
♦ Excluded from analysis (give reasons) (n= 0)

Randomized (n= 90)
